# The relationship between motivation for having children and sociodemographic and obstetric characteristics: a cross-sectional study

**DOI:** 10.1186/s12884-025-07956-8

**Published:** 2025-08-14

**Authors:** Zümrüt Bilgin, İrem Özten Dalkıran, Esra Sarı, Khaled Trabelsi, Waqar Husain, Achraf Ammar, Haitham Jahrami

**Affiliations:** 1 Faculty of Health Science, Department of Midwifery, Marmara University, 34854 Istanbul, Türkiye; 2 Enstitute of Health Sciences, University of Health Sciences, 34668 Istanbul, Türkiye; 3 Department of Midwifery, Faculty of Health Science, Van Yuzuncu Yil University, 65080 Van, Türkiye; 4 Research Laboratory Education, Motricité, Sport et Santé, EM2S, LR19JS01, High Institute of Sport and Physical Education of Sfax, University of Sfax, Sfax, Tunisia; 5 Department of Movement Sciences and Sports Training, School of Sport Science, The University of Jordan, Amman, Jordan; 6 Department of Humanities, COMSATS University Islamabad, Park Road, Islamabad, Pakistan; 7 Department of Training and Movement Science, Institute of Sport Science, Johannes Gutenberg-University Mainz, Mainz, Germany; 8 Research Laboratory, Molecular Bases of Human Pathology, LR19ES13, Faculty of Medicine of Sfax, University of Sfax, 3000 Sfax, Tunisia; 9 Department of Psychiatry, College of Medicine and Health Sciences, Arabian Gulf University, Manama, 329 Bahrain; 10 Government Hospitals, Manama, Bahrain

**Keywords:** Childbearing, Motivation, Obstetric characteristics, Sociodemographic characteristics

## Abstract

**Objective:**

This study aims to examine the relationships between motivation for having children and sociodemographic and obstetric characteristics.

**Materials and methods:**

This descriptive, cross-sectional study was conducted between February 2025 and March 2025. Data were collected via the “Personal Information Form” and the “Motivations for Having Children Scale”. The data of 185 women who participated in the study were analyzed, and the statistical significance level was accepted as *p* < 0.05.

**Results:**

The mean age of the women who participated in the study was 32.94 ± 7.63 years; 65.4% were university graduates, and 42.2% wanted to have children. A significant correlation was found between the desire for children and age, education level, income level, social support status and duration of marriage (*p* < 0.05). In the present study, 27.6% of the women had one pregnancy, 31.4% had living children, and a relationship was found between the number of pregnancies, the number of living children and the time elapsed since the last pregnancy. In this study, there was no significant difference between the total score of the Motivation to Have Children Scale and age, educational level, income level, or duration of marriage (*p* > 0.05), whereas a significant difference was found between employment status and spousal compatibility (*p* < 0.05). The mean total score of the Motivation to Have Children Scale was calculated as 92.38 ± 26.31.

**Conclusion:**

According to the findings of the study, it was determined that women’s employment status and spousal adjustment affected women’s motivation to have children. In this direction, it is thought that psychosocial factors, in addition to sociodemographic and obstetric characteristics, will play a critical role in determining reproductive motivations. Understanding these factors may help reproductive health professionals and policymakers develop interventions for women’s motivation to have children and support family planning efforts in different social contexts.

## Introduction

In all cultures, fertility is considered fundamental, representing the ultimate ideal of life and a highly desirable objective [1]. A primary aspiration for married individuals is the desire to conceive and raise children [2]. The decision to have children is influenced by a variety of factors, including the motivation to parent, which significantly impacts women’s choices regarding childbearing [3].

The sociodemographic characteristics of couples and the obstetric characteristics of women significantly influence the desire and motivation to have children. Sociodemographic factors, including age, gender, education level, and income, play a crucial role in the decision-making processes of individuals [4]. Additionally, women’s obstetric characteristics, such as experiences related to pregnancy and childbirth, are also influential. A study identified statistically significant differences in fertility motivations based on variables such as age, education level, age at marriage, duration of marriage, occupation, spouse’s education level, family size, and monthly income status [5]. Furthermore, a review of the literature indicates that motivations for childbearing can vary widely among women, with some exhibiting high motivations while others display low motivations [6, 7]. In a study by Ahmed et al. (2022) involving 1,085 married women, it was found that 42.1% expressed a positive desire to have children, while 45% indicated a negative desire [5].

Unplanned pregnancies directly impact maternal and child health. Women’s childbearing motivations are also important in terms of planning their pregnancies. Women need family planning methods to plan their pregnancies. However, most women do not use family planning methods for various reasons. This situation results in unplanned and/or unintended pregnancies [8]. According to the 2018 Türkiye Demographic and Health Survey data, 75% of the births in the five years preceding the survey occurred at the desired time, 11% would have preferred a later time, and 15% did not desire a pregnancy at all [9]. Unplanned and/or unintended pregnancies lead to inadequate or absent prenatal care, a lack of folic acid supplementation, and increased anxiety and depression [10, 11]. It is known that women’s thoughts about fertility have been significantly affected by the increase in the level of education in Türkiye in recent years. It is essential for reproductive health specialist to determine the factors affecting the motivation of women of reproductive age to have children, to develop fertility awareness and to plan reproductive health services. The aim of this study was to determine the relationships between childbearing motivation and sociodemographic and obstetric characteristics.

While previous studies have examined fertility motivations in various contexts, there is a notable lack of research investigating the combined effects of sociodemographic and obstetric characteristics on childbearing motivation, particularly in Türkiye. The existing literature frequently emphasizes isolated factors such as age or education, with insufficient attention paid to the interaction of psychosocial and obstetric variables in shaping reproductive decisions. This study addresses this gap by utilizing the validated Childbearing Motivation Scale to explore how sociodemographic factors (e.g., age, education, income) and obstetric characteristics (e.g., number of pregnancies, planning status) interact to influence women’s fertility motivations within a hospital-based sample in Turkey. By focusing on these factors in the context of Tekirdag, Türkiye, this research offers region-specific insights that can inform targeted reproductive health interventions and family planning policies.

*Research questions*.

### RQ_1_

Is there a statistically significant relationship between women’s sociodemographic characteristics and their childbearing motivations?

### RQ_2_

Is there a statistically significant relationship between women’s obstetric characteristics and their childbearing motivations?

### RQ_3_

Do childbearing motivation scores differ significantly according to women’s sociodemographic and obstetric characteristics?

## Materials and methods

### Type, place and time of the study

This descriptive, cross-sectional study was conducted at a hospital in Tekirdag Province between February 2025 and March 2025.

### Population and sample of the study

The population of the study consisted of women who applied to the gynecology and obstetrics clinic of a hospital in Tekirdag province. The sample of the study consisted of women who applied to the gynecology and obstetrics clinic between February 2025 and March 2025 and who voluntarily accepted to participate in the study. Before starting the study, the sample size was calculated with the G*Power 3.1 program. In order to exceed 95% in determining the power of the test, it was aimed to reach 176 people at a significance level of 5% and an effect size of 0.50. Post hoc sampling calculation was performed for 185 participants before data analysis. With an effect size of 0.50, a standard error of 0.05 and a confidence interval of 0.99, the sample size of the study was found to be sufficient.

### Data collection tools

The data of the study were collected through face‒to‒face interviews in a setting where women’s privacy was protected, using the “Personal Information Form” created by the researchers and the “Childbearing Motivation Scale” within 15–20 min.

#### Personal information form

The form was developed by the researchers on the basis of the relevant literature [2–12]. The form consists of 15 questions regarding women’s sociodemographic characteristics (age, education level, employment status, perceived income, perceived social support, etc.), obstetric characteristics (number of pregnancies, planning status of the most recent pregnancy, reasons for desiring or not desiring to have children, etc.), and pregnancy intentions.

#### Childbearing motivation scale (CMS)

The Turkish validity and reliability study of the scale developed by Guedes et al. to determine individuals’ motivations for having or not having children was conducted by Huseyinzade Simsek [13, 14]. The Childbearing Motivation Scale consists of positive childbearing motivations (22 items, 3 factors) and negative childbearing motivations (13 items, 3 factors) subscales and is a five-point Likert-type scale ranging from “not at all” to “completely”. The positive childbearing motivations subscale consists of socioeconomic views, couple relationships and personal satisfaction factors, while the negative childbearing motivations subscale consists of marital stress, financial problems and social and ecological concerns. When scoring the positive items, the answer “not at all” is scored with “1” and the answer “completely” is scored with “5”. The high scores obtained from this subscale indicate that individuals find these statements very important for being a mother/father. When scoring the negative items, the response “not at all” is scored as “1” and the response “completely” is scored as “5”. High scores obtained from the statements of this subscale enable individuals to express that they have important reasons for not being a mother/father. The lowest score that can be obtained from the whole scale is 35 and the highest score is 175. Cronbach’s Alpha reliability coefficient was found to be 0.916 [14] In this study, the Cronbach’s alpha coefficient of the scale was calculated as 0.921.

### Evaluation of the study data

For the statistical analysis, the frequency (n) and percentage (%) values and the mean (Mean), standard deviation (SD), minimum (Min), and maximum (Max) values were calculated.

The Shapiro‒Wilk test was used to evaluate whether the data were normally distributed. For data that did not meet the assumption of a normal distribution, the Mann‒Whitney *U* test was used for comparisons between two independent groups, and the Kruskal‒Wallis *H* test was used for comparisons between three or more independent groups. A Spearman correlation test was conducted. In the evaluation of the hypothesis test findings obtained in the study, the margin of error was set at 5%. The hypothesis test findings were obtained via the IBM Statistical Package for the Social Sciences, Version 26.0 (IBM SPSS 26.0) program.

## Results

The mean age of the women who participated in the study was 32.94 ± 7.63 years, with the youngest being 19 years old and the oldest being 49 years old. In the present study, 42.2% of the women desired to have children, whereas 57.8% did not desire to have children. A total of 65.4% of the women were university graduates, 73% were not employed, 72.4% had an income equal to their expenses, 94.1% lived in a nuclear family, 88.1% had sufficient social support, 91.4% had spousal harmony, and 41.1% had been married for 5 years. There was a significant relationship between the desire to have children and age, education level, income level, social support status, spousal harmony, and duration of marriage (*p* < 0.05) (Table 1).

**Table 1:** Sociodemographic characteristics of women (*n* = 185, Türkiye, 2025)

Characteristics	Desire to Have Children						X^2^ and *p* Value
	Yes (78)		No (107)		Total		
	*n*	%	*n*	%	*n*	%	
*Age--Year* (Mean = 32.94 ± 7.63; Min = 19; Max = 49)							
Aged 18–29 years	51	65.4	21	19.6	72	38.9	45.796 **0.000**
Aged 30–39 years	23	29.5	47	43.9	70	37.8	
40 years and above	4	5.1	39	36.4	43	23.2	
*Education level*							
Primary + Secondary School	4	5.1	15	14.0	19	10.3	8.461 **0.015**
High school	14	17.9	31	29.0	45	24.3	
University	60	76.9	61	57.0	121	65.4	
*Employment status*							
Employed	62	79.5	73	68.2	135	73.0	2.902 0.088
Unemployed	16	20.5	34	31.8	50	27.0	
*Income level*							
Income less than expenses	6	7.7	7	6.5	13	7.0	9.164 **0.010**
Income equal to expenses	48	61.5	86	80.4	134	72.4	
Income more than expenses	24	30.8	14	13.1	38	20.5	
*Family type*							
Nuclear	76	97.4	98	91.6	174	94.1	2.758 0.097
Extended	2	2.6	9	8.4	11	5.9	
*Social support status (spouse, family support)*							
Adequate	74	94.9	89	83.2	163	88.1	5.888 **0.015**
Inadequate	4	5.1	18	16.8	22	11.9	
*Spousal harmony*							
Yes	76	97.4	93	86.9	169	91.4	6.319 **0.012**
No	2	2.6	14	13.1	16	8.6	
*Duration of marriage*							
0–5 years	56	71.8	20	18.7	76	41.1	64.056 **0.000**
6–11 years	16	20.5	22	20.6	38	20.5	
≥ 12 years	6	7.7	65	60.7	71	38.4	

χ² represents the chi-square test used to evaluate the association between sociodemographic characteristics and the desire to have children. A p-value < 0.05 indicates statistical significance

It was determined that 27.6% of the women were experiencing their first pregnancy, 20.5% had experienced a miscarriage, 31.4% had living children, 70.8% had planned their last pregnancy, 30.3% had a time gap of 1–3 years since their last pregnancy, 22.7% had a vaginal delivery, and 61.6% were using contraceptive methods. There was a relationship between the desire to have children and the number of pregnancies, the number of living children, pregnancy planning status, the time elapsed since the last pregnancy, the mode of delivery, and the use of contraceptive methods (*p* < 0.05) (Table 2).

**Table 2:** Obstetric characteristics of women (*n* = 185, Türkiye, 2025)

Characteristics	Desire to Have Children						X^2^ and *p* Value
	Yes (78)		No (107)		Total		
	*n*	%	*n*	%	*n*	%	
*Number of pregnancies*							
First pregnancy	28	35.9	23	21.5	51	27.6	45.319 **0.000**
≥ 2 Pregnancies	16	20.5	73	68.2	89	48.1	
No pregnancy	34	43.6	11	10.3	45	24.3	
*Miscarriage*							
Yes	11	14.1	27	25.2	38	20.5	3.425 0.064
No	67	85.9	80	74.8	147	79.5	
*Number of living children*							
1 child	27	34.6	31	29.0	58	31.4	52.288 **0.000**
≥ 2 children	10	12.8	64	59.8	74	40.0	
No children	41	52.6	12	11.2	53	28.6	
*Time elapsed since the last pregnancy*							
1–3 years	30	38.5	26	24.3	56	30.3	51.323 **0.000**
≥ 4 years	12	15.4	70	65.4	82	44.3	
No pregnancy	36	46.2	11	10.3	47	25.4	
*Planning status of the last pregnancy*							
Planned	44	56.4	87	81.3	131	70.8	31.088 **0.000**
Unplanned	0	0.0	9	8.4	9	4.9	
No pregnancy	34	43.6	11	10.3	45	24.3	
*Mode of the last delivery*							
Vaginal delivery	10	12.8	32	29.9	42	22.7	37.928 **0.000**
Cesarean Section	26	33.3	62	57.9	88	47.6	
No childbirth	42	53.8	13	12.1	55	29.7	
*Use of contraceptive method*							
Yes	40	51.3	74	69.2	114	61.6	6.096 **0.014**
No	38	48.7	33	30.8	71	38.4	

χ² represents the chi-square test used to evaluate the association between sociodemographic characteristics and the desire to have children. A p-value < 0.05 indicates statistical significance

In this study, no significant difference was found between the total score of the Childbearing Motivation Scale and age, education level, income level, or duration of marriage (*p* > 0.05), whereas a significant difference was found between employment status and spousal harmony (*p* < 0.05) (Table 3).

**Table 3:** Comparison of women’s sociodemographic characteristics with CMS mean score (*n* = 185, Türkiye, 2025)

Characteristics	*n* (185)	50th (Median)	25th −75th	Statistics and *P* Value
*Age*				*χ² and p*
Aged 18–29 years	72	89.00	77.00-105.75	0.062 0.970
Aged 30–39 years	70	86.50	74.00-112.50	
≥ 40 years	43	86.00	72.00-112.00	
*Education level*				*χ² and p*
Primary + Secondary School	19	91.00	86.00-109.00	1.304 0.521
High school	45	89.00	73.50-115.50	
University	121	86.00	75.00-106.50	
*Employment status*				*Z and p*
Employed	135	85.00	72.00-109.00	−2.274 **0.023**
Unemployed	50	91.00	85.75–109.00	
*Income status*				*χ² and p*
Income less than expenses	13	89.00	73.00-102.50	2.156 0.340
Income equal to expenses	134	89,50	78.75–110.50	
Income less than expenses	38	81.00	71.75-105.25	
*Family type*				*Z and p*
Nuclear	174	87.50	75.00-108.25	−0.738 0.461
Extended	11	86.00	85.00-115.00	
*Social support status (spouse, family support)*				*Z and p*
Adequate	163	87.00	75.00-109.00	−0.136 0.892
Inadequate	22	87.50	74.00-116.00	
*Spousal harmony*				*Z and p*
Yes	169	89.00	77.50–111,00	−2.450 **0**.**014**
No	16	76.00	61.00–90,50	
*Duration of marriage*				*χ² and p*
0–5 years	76	86.00	75.25-100.75	2.695 0.260
6–11 years	38	93.50	79.50-118.25	
≥ 12 years	71	87.00	72.00-113.00	

*CMS* Childbearing Motivation Scale. χ² represents the Kruskal-Wallis test for comparisons across three or more groups (age, education level, income status, duration of marriage). Z represents the Mann-Whitney *U* test for comparisons between two groups (employment status, family type, social support status, spousal harmony). A p-value < 0.05 indicates statistical significance

In this study, no significant differences were found between the total score of the Childbearing Motivation Scale and the number of pregnancies, the number of living children, the planning status of the last pregnancy, the time elapsed since the last pregnancy, the mode of the last delivery, or the use of contraceptive methods (*p* > 0.05) (Table 4).

**Table 4:** Comparison of women’s obstetric characteristics with CMS mean score (*n* = 185, Türkiye, 2025)

Characteristics	*n* (185)	50th (Median)	25th −75th	Statistics and *P* Value
*Number of pregnancies*				χ² *and p*
First pregnancy	51	86.00	72.00-108.00	1,075 0,584
≥ 2 Pregnancies	89	88.00	75.00-117.00	
No pregnancy	45	90.00	79.00-102.00	
*Number of living children*				χ² *and p*
1 child	58	85.50	71.75-112.25	0,615 0,735
≥ 2 children	74	88.00	74.75–116.50	
No children	53	88.00	79.00-99.50	
*Planning status of the last pregnancy*				χ² *and p*
Planned	131	86.00	74.00-112.00	0,048 0,976
Unplanned	9	86.00	76.50–113.00	
No pregnancy	45	90.00	79.00-102.00	
*Time elapsed since the last pregnancy*				χ² *and p*
1–3 years	57	85,00	71.00-108.00	1,412 0,494
≥ 4 years	83	89.00	78.00-115.00	
No pregnancy	45	90.00	79.00-102.00	
*Mode of the last delivery*				χ² *and p*
Vaginal delivery	42	93.50	82.00-126.25	6,002 0,050
Cesarean Section	88	86.00	71.00-109.00	
No childbirth	55	87.00	79.00–99.00	
*Use of contraceptive methods*				Z and p
Yes	114	86.50	75.00-109.75	−0.097 0.922
No	71	89.00	78.00-107.00	

*CMS *Childbearing Motivation Scale. χ² represents the Kruskal-Wallis test for comparisons across three or more groups (number of pregnancies, number of living children, planning status of the last pregnancy, time elapsed since the last pregnancy, mode of the last delivery). Z represents the Mann-Whitney *U* test for comparisons between two groups (use of contraceptive methods). A p-value < 0.05 indicates statistical significance

The mean total score of the Childbearing Motivation Scale was 92.38 ± 26.31 (min–max: 35–175). The mean total score of the Positive Childbearing Motivation Scale was 60.09 ± 20.30 (min–max: 22–110), and the mean total score of the Negative Childbearing Motivation Scale was 32.29 ± 13.40 (min-max: 13–65).

A weak positive correlation was found between the total score of the Childbearing Motivation Scale and employment status (*r* = 0.168, *p* = 0.023), whereas a weak negative correlation was found between the total score of the Childbearing Motivation Scale and spousal harmony (*r* = −0.181, *p* = 0.014). A weak positive correlation was found between the total score of the Negative Childbearing Motivation Scale and employment status (*r* = 0.146, *p* = 0.047), whereas a weak negative correlation was found between the total score of the Positive Childbearing Motivation Scale and spousal harmony (*r* = −0.184, *p* = 0.012).

## Discussion

In this study, which aimed to determine the relationships between childbearing motivation and sociodemographic and obstetric characteristics, the mean age of the women was 32.94 ± 7.63 years. In a study conducted by Azmoude et al. (2017), the mean age of the women was determined to be 31.12 ± 6.61 years [15]. In a study conducted by Irani and Khadivzadeh (2018), the mean age of the women was determined to be 31.5 years, and 49.5% of the women were between the ages of 25 and 35 [3]. The results of this study are consistent with the findings of other studies.

According to the findings of this study, 65.4% of the women were university graduates, 73% were not employed, and 72.4% had an income equal to their expenses. In a study conducted by Zare, Kiaetabar, and Ahangar (2019) in Iran with 450 women aged 18–35, 36% of the women had an associate or bachelor’s degree, 86.4% were housewives, and 72.4% had an average income status [16]. There is a difference between the conducted study and the results of this study in terms of education levels. This difference is thought to stem from the region where the studies were conducted and the country’s education policies.

Starting a family is one of the most exciting and important periods in an individual’s life. Many people dream of having children after starting a family [17]. According to the findings of this study, 61.6% of the women used contraceptive methods, whereas a study conducted by Kamiloglu and Irmak Vural (2022) reported that 86.7% of the women used family planning methods [18]. Today, the use of family planning methods has transformed the likelihood of parenthood into a choice [19]. In this study, 57.8% of the women did not desire to have children. A study conducted by Azmoude et al. (2020) revealed that 52.7% of women had no fertility intentions [15]. According to the 2018 Türkiye Demographic and Health Survey data, 53% of currently married women did not desire to have more children, whereas 14% stated that they desired their next pregnancy to be at least two years later [9]. The rise of contraception, education and greater participation in the labor market are among the reasons for fertility postponement and decline [19, 20]. In terms of the status of having children, the results of this study are consistent with the literature. However, in terms of planning the next pregnancy, the results of this study differ from the 2018 Türkiye Demographic and Health Survey data. This difference is thought to be caused by economic and social factors.

A weak positive correlation was found between the total score of the Childbearing Motivation Scale and employment status (*r* = 0.168, *p* = 0.023), whereas a weak negative correlation was found between the total score of the Childbearing Motivation Scale and spousal harmony (*r* = −0.181, *p* = 0.014). A weak positive correlation was found between the total score of the Negative Childbearing Motivation Scale and employment status (*r* = 0.146, *p* = 0.047), whereas a weak negative correlation was found between the total score of the Positive Childbearing Motivation Scale and spousal harmony (*r* = −0.184, *p* = 0.012). In the study conducted by Ustun and Beydag (2024), it was found that the mean score of the “negative impact of having children on life” sub-dimension of women who were employed was higher than other women, while the mean score of the “positive impact of having children on life” sub-dimension of women who were not employed was higher [21]. In other words, it can be said that the desire of working women to have children is lower than non-working women. The idea that having children may interfere with the career life of working women, the lack of facilities such as kindergartens in workplaces, and the fact that employers prefer to employ women without children or single women rather than pregnant women or women with children may cause women to limit the number of children. In addition to these issues, difficulties experienced at work make childcare more difficult for women.

In a study conducted by Zare, Kiaetabar, and Ahangar (2019) in Iran, a relationship was found between education level and positive fertility motivation, whereas a significant relationship was found between income status and negative fertility motivation [16]. In a study conducted by Irani and Khadivzadeh (2018) with 844 women of reproductive age (15–49 years) who were marrying for the first time in various regions of Mashhad, positive and negative childbearing motivations were not related to the actual number of children [3]. A review of the literature has revealed that better career expectations increase the desire to have children in both men and women [22–24]. The results of this study differ from the findings of the studies conducted by Zare, Kiaetabar, and Ahangar but are similar to the results of the studies conducted by Irani and Khadivzadeh. In a study conducted by Ciritel, De Rose, and Arezzo (2019) in Romania, which has a low fertility rate, age was identified as the only variable that had a significant effect on the intention to have children for both childless individuals and parents with one child [25]. The results of this study differ from the findings of studies conducted by Critel, De Rose, and Arezzo. The difference between the two studies is thought to stem from the difference in the perception of childbearing.

This study identified weak correlations between childbearing motivation and specific sociodemographic factors, offering nuanced insights into their influence. A weak positive correlation (*r* = 0.168, *p* = 0.023) between employment status and the total score of the Childbearing Motivation Scale indicates that employed women tend to exhibit slightly higher childbearing motivation, potentially due to increased financial stability or access to resources that facilitate family planning. Conversely, a weak negative correlation (*r* = −0.181, *p* = 0.014) between spousal harmony and the total score suggests that women with lower spousal compatibility may experience diminished motivation for childbearing, possibly reflecting relational stressors that affect fertility decisions. Similarly, the weak positive correlation (*r* = 0.146, *p* = 0.047) between employment status and the Negative Childbearing Motivation Scale implies that employed women may perceive greater barriers to parenthood, such as challenges related to work-life balance. In contrast, the weak negative correlation (*r* = −0.184, *p* = 0.012) with the Positive Childbearing Motivation Scale and spousal harmony emphasizes the significance of relationship quality in fostering positive fertility intentions. These findings underscore the complex interplay of psychosocial factors in shaping reproductive motivations within the Turkish context.

## Conclusion

The study revealed a significant relationship between the desire to have children and sociodemographic variables such as age, education level, and income level, as well as obstetric variables such as the number of pregnancies and the number of living children. A significant difference was found between the total score of the Childbearing Motivation Scale and women’s employment status and spousal harmony. To increase childbearing motivation, it may be necessary to develop multidimensional programs tailored to the characteristics of each region and/or geographical area. It is also recommended that large-sample studies examining the factors that influence childbearing motivation be conducted.

## Data Availability

Availability of data and material: The original contributions presented in the article; further inquiries can be directed to the first author.
